# Effectiveness of a Web-Based Menu-Planning Intervention to Improve Childcare Service Compliance With Dietary Guidelines: Randomized Controlled Trial

**DOI:** 10.2196/13401

**Published:** 2020-02-04

**Authors:** Alice Grady, Luke Wolfenden, John Wiggers, Chris Rissel, Meghan Finch, Victoria Flood, David Salajan, Ruby O'Rourke, Fiona Stacey, Rebecca Wyse, Christophe Lecathelinais, Courtney Barnes, Sue Green, Vanessa Herrmann, Sze Lin Yoong

**Affiliations:** 1 School of Medicine and Public Health University of Newcastle Callaghan Australia; 2 Population Health Hunter New England Local Health District Wallsend Australia; 3 Hunter Medical Research Institute New Lambton Australia; 4 Priority Research Centre for Health Behaviour University of Newcastle Callaghan Australia; 5 School of Public Health Faculty of Medicine and Health The University of Sydney Camperdown Australia; 6 New South Wales Office of Preventive Health Liverpool Australia; 7 Westmead Hospital Western Sydney Local Health District Westmead Australia; 8 Faculty of Health Sciences and Charles Perkins Centre The University of Sydney Sydney Australia; 9 Healthy Australia Ltd Melbourne Australia

**Keywords:** child care, child, preschool, online systems, menu planning, nutrition policy, randomized controlled trial, internet-based intervention

## Abstract

**Background:**

Foods provided in childcare services are not consistent with dietary guideline recommendations. Web-based systems offer unique opportunities to support the implementation of such guidelines.

**Objective:**

This study aimed to assess the effectiveness of a Web-based menu planning intervention in increasing the mean number of food groups on childcare service menus that comply with dietary guidelines. Secondary aims were to assess the impact of the intervention on the proportion of service menus compliant with recommendations for (1) all food groups; (2) individual food groups; and (3) mean servings of individual food groups. Childcare service use and acceptability of the Web-based program were also assessed.

**Methods:**

A single-blind, parallel-group randomized controlled trial was undertaken with 54 childcare services in New South Wales, Australia. Services were randomized to a 12-month intervention or usual care control. Intervention services received access to a Web-based menu planning program linked to their usual childcare management software system. Childcare service compliance with dietary guidelines and servings of food groups were assessed at baseline, 3-month follow-up, and 12-month follow-up.

**Results:**

No significant differences in the mean number of food groups compliant with dietary guidelines and the proportion of service menus compliant with recommendations for all food groups, or for individual food groups, were found at 3- or 12-month follow-up between the intervention and control groups. Intervention service menus provided significantly more servings of fruit (*P*<.001), vegetables (*P*=.03), dairy (*P*=.03), and meat (*P*=.003), and reduced their servings of discretionary foods (*P*=.02) compared with control group at 3 months. This difference was maintained for fruit (*P*=.03) and discretionary foods (*P*=.003) at 12 months. Intervention childcare service staff logged into the Web-based program an average of 40.4 (SD 31.8) times and rated the program as highly acceptable.

**Conclusions:**

Although improvements in childcare service overall menu and individual food group compliance with dietary guidelines were not statistically significant, findings indicate that a Web-based menu planning intervention can improve the servings for some healthy food groups and reduce the provision of discretionary foods. Future research exploring the effectiveness of differing strategies in improving the implementation of dietary guidelines in childcare services is warranted.

**Trial Registration:**

Australian New Zealand Clinical Trial Registry (ANZCTR): 16000974404; http://www.anzctr.org.au/ACTRN12616000974404.aspx

## Introduction

### Background

Poor diet is a modifiable risk factor for the development of noncommunicable diseases including stroke, diabetes, and heart disease, accounting for 19% mortality and 10% of morbidity, globally [1]. Population surveys in Australia and internationally indicate that both adults and young children are not consuming the recommended servings of fruit and vegetables and consume more than recommended amounts of discretionary (energy-dense and nutrient-poor) foods [2-5]. As dietary behaviors established in early childhood track into adulthood [6,7], the World Health Organization recommends that population health approaches be undertaken to improve healthy eating behaviors in young children [8,9].

As approximately 662,000 children aged 0 to 5 years attend formal care in Australia [10], childcare services represent an opportune environment in which to intervene to establish healthy eating behaviors. Systematic review evidence, leading health authorities, and governments internationally recommend that childcare services provide foods in line with dietary guidelines [2,8,11-14]. In the state of New South Wales (NSW), Australia, the Caring for Children [15] resource outlines best practice dietary guidelines for the childcare sector. However, research internationally and in Australia suggests that such dietary guidelines are poorly implemented, with childcare services frequently providing foods and drinks inconsistent with guideline recommendations [16-19].

Childcare staff have reported a number of barriers to the implementation of dietary guidelines. Findings from a recent systematic review indicated such barriers to childcare service staff implementation of guidelines related to knowledge, skills, social influences, environmental context, and a lack of resources [20]. These barriers center around the lack of staff training and support to undertake menu planning consistent with guidelines and regulatory standards (eg, child allergies) and challenges associated with self-assessment of a menu to determine the nutritional adequacy [18,21-24] and its compliance with guidelines.

To improve the implementation of dietary guidelines in childcare, strategies that target known barriers to implementation are required. To our knowledge, only 4 controlled trials have been conducted with the aim of improving the provision of foods and beverages to children in childcare in accordance with dietary guidelines [17,19,25,26]. All 4 trials assessed the impact of multistrategy interventions consisting of a combination of educational materials, face-to-face meetings, or audit and feedback; and when compared with control groups, none found significant improvements in the implementation of the targeted dietary guidelines. The implementation support strategies utilized in these previous trials, therefore, appear insufficient to address knowledge and skill barriers to the implementation of dietary guidelines in this setting.

Web-based interventions offer an opportunity to provide implementation support that has the potential to be effective in enhancing childcare service implementation of dietary guidelines. First, childcare services have existing infrastructure (computer and internet access) to support a Web-based intervention [27]; and staff are willing to use such an intervention to support their implementation of healthy eating policies and practices [27]. Second, specific programming within Web-based systems [28] has the potential to integrate active behavior change strategies [29] to target primary barriers to guideline implementation, including resources, audit, and feedback for menus, and automated calculation of menu compliance with guidelines, eliminating the need for manual calculations by service staff. Third, Web-based interventions can be tailored to a particular service’s needs, delivered with high fidelity, at low end-user cost, and are able to address equity issues related to access to dietetic support, particularly for childcare services in rural and remote areas [30,31]. Finally, Web-based systems have the potential to minimize the need for ongoing investment in implementation support (eg, the provision of training and resources) for practice improvements to be sustained.

### Objectives

Despite this potential, the effectiveness of a Web-based intervention to improve childcare service implementation of dietary guidelines has not yet been evaluated [32]. As such, the primary aim of the study was to assess, compared with usual care, the effectiveness of a Web-based menu planning intervention in increasing the mean number of food groups on childcare service menus that comply with dietary guidelines. Secondary aims include assessment of the impact of the intervention on the proportion of service menus compliant with (1) all food groups; (2) individual food groups; and (3) the mean servings of individual food groups. Childcare service use and acceptability of the Web-based program were also assessed.

## Methods

### Ethics Approval and Consent to Participate

Ethical approval was obtained from the Hunter New England (approval no: 16/02/17/4.05) and the University of Newcastle (approval H-2016-0111) Human Research Ethics Committees. The trial was prospectively registered with the Australian New Zealand Clinical Trials Registry (ACTRN12616000974404). Other registered secondary outcomes will be reported elsewhere. The reporting of this study adheres to the Consolidated Standards of Reporting Trials guidelines [33]. All subjects in this research study provided consent to participate.

### Design and Setting

As previously described in the study protocol [34], a parallel-group randomized controlled trial (RCT) was undertaken with 54 long day care services in NSW, Australia. The 252 potentially eligible childcare services in NSW that were current clients of a single specific childcare management software (CCMS) provider, and that provided foods to children, served as the study sampling frame. In order for families to receive financial reimbursement from the Australian government to assist with the costs of childcare [35], services are mandated by Federal legislation to use a government-approved CCMS. The Web-based intervention, titled *feedAustralia*, was developed by Hubcare Innovation, for Healthy Australia and in collaboration with HubHello, and was linked to one such software package used by approximately 20% of childcare services in NSW [36].

### Participants

Eligible childcare services were required to (1) be open for ≥8 hours each weekday; (2) prepare and provide at least 1 main meal and 2 snacks to children onsite each weekday; (3) have service staff make menu planning decisions; and (4) have a menu planner with sufficient English to engage with the intervention. Services that outsourced menu planning, did not cater for children aged 3-6 years, catered exclusively for special needs children, or were run by the NSW Department of Education were excluded because of differing administrative characteristics.

### Recruitment

All services in the sampling frame were posted an invitation letter and information statements about the study in random order, approximately 2 weeks before receiving a call from a research assistant to assess eligibility and obtain service consent to participate (August-December 2016). Recruitment of services was conducted in random order as a subsample of services also participated in a nested evaluation [34]. The CCMS provider also displayed an invitation for services to participate in the trial via their Web access portal. Following provision of consent, nominated supervisors and menu planners were contacted to complete a computer-assisted telephone interview (CATI) to assess baseline service and menu planner characteristics and were asked to provide a 1-week-long menu from their current menu cycle for assessment.

### Randomization and Allocation

Following the completion of baseline data collection, services were allocated to the intervention or control group in a 1:1 ratio, stratified by service area socioeconomic status (as determined by service postcode) [37] by an independent statistician using a random number function in Microsoft Excel 2010. All outcome data assessors were blind to group allocation; however, owing to the nature of the trial, childcare staff and health promotion officers delivering the intervention were aware of group allocation.

### Intervention

Services received a 12-month implementation intervention consisting of access to a Web-based menu planning program (*feedAustralia*), in addition to training and support to use the program (Multimedia Appendix 1 [15,28,34,38-44]). The menu planning program was not embedded within the CCMS platform already used by the childcare services as originally planned because of changes in national regulatory requirements for CCMS. Rather, the menu planning program was developed as a stand-alone program, allowing childcare services to access the program outside of CCMS. The program was linked to the Web-based CCMS platform to allow communication between the 2 systems. The intervention was codeveloped and overseen by an experienced multidisciplinary expert advisory group consisting of health promotion practitioners, implementation and behavioral scientists, policy makers, and public health nutritionists with experience working in the setting. To ensure uptake and to enhance use of the Web-based program, the menu planning program was developed using the Technology Acceptance Model [45], with implementation support strategies identified through a barriers assessment using the Theoretical Domains Framework [46]. Further details regarding the theoretical underpinnings and development of the intervention are reported elsewhere [34].

### Control Group

Services randomly allocated to the control group did not receive access to the Web-based menu planning program or other implementation support strategies.

### Data Collection Procedures and Measures

Baseline data were collected during October 2016 to April 2017, with the 12-month follow-up conducted during October 2017 to March 2018.

#### Primary Outcome: Mean Number of Food Groups Compliant With Dietary Guidelines

As a summary indicator of childcare service menu compliance, the primary outcome was the mean number of food groups on the menu that were compliant with dietary guidelines for the sector [15] at the 12-month follow-up. The majority of childcare services in NSW typically plan their menus in cycles of 2 to 6 weeks [18]. As such, at baseline, 3-month follow-up, and 12-month follow-up, a dietitian or nutritionist blinded to service allocation randomly selected 1 week of each services’ current menu cycle for review to eliminate selection bias, using the random number function in Microsoft Excel 2010. Menus were assessed using best practice protocols [47] to calculate the number of servings of each food group that the menu provided per child, per day.

Dietary guidelines for the setting [15] recommend that services provide the following servings at a minimum, of each of the following Australian Guide to Healthy Eatin (AGHE) [14] food groups on a daily basis for children in care for 8 hours: (1) vegetables and legumes/beans (2 servings); (2) fruit (1 serving); (3) wholegrain (cereal) foods and breads (2 servings); (4) lean meat and poultry, fish, eggs, tofu, seeds, and legumes (3/4 serving); (5) milk, yoghurt, cheese, and alternatives (1 serving); and (6) no *discretionary* foods that are high in energy and low in nutrients (0 servings). A food group was only considered compliant when the minimum recommended number of servings, and no discretionary foods, were provided for every child, every day over a 1-week period. A menu was only considered compliant when the minimum recommended number of servings of all food groups, and no discretionary foods, were provided for every child, every day over a 1-week period.

#### Secondary Outcomes

The secondary outcomes were as follows:

- Compliance with dietary guidelines for all food groups: To identify absolute compliance with dietary guidelines, the proportion of services compliant for all of the 6 food groups was assessed via 1-week menu review at baseline, 3-month follow-up, and 12-month follow-up.
- Individual food group compliance with dietary guidelines: To identify variation in compliance with dietary guidelines for individual food groups, the proportion of services compliant with dietary guidelines for each of the 6 food groups individually was compared between the intervention and control groups as assessed via 1-week menu review at baseline, 3-month follow-up, and 12-month follow-up.
- Mean servings of individual food groups: To identify any changes in the quantities or times an individual food group was provided on the menu, an additional exploratory outcome was included. This measure was not prospectively registered. The mean number of servings for each of the 5 food groups (vegetables, fruit, breads and cereals, meat and dairy) and the number of times discretionary foods were provided on the menu daily were compared between the intervention and control groups as assessed via 1-week menu review (resulting in 5 days of data per service) at baseline, 3-month follow-up, and 12-month follow-up.

#### Other Data

A range of other data were assessed as follows:

- Service and menu planner characteristics: Nominated supervisors and menu planners completed a CATI at baseline to obtain service postcode (to determine service area socioeconomic status and geographic location), whether any children of aboriginal and/or Torres Strait Islander background were enrolled, the number of children attending each day, service hours of operation, and menu planner age, qualifications and years working as a service cook. Items have been used previously by the research team in surveys conducted with childcare services [18,20].
- Use of the Web-based program: Google Analytics data [48] routinely collected by the CCMS provider were used to assess service engagement with the menu planning program at the 12-month follow-up. This included the frequency of access, number of times key features were accessed (menu, recipes, nutrition checklist, analytics, and guidelines), and the number of helpdesk queries made in relation to the program.
- Intervention delivery: Internal records maintained by the project team were used to monitor the delivery of the intervention support.
- Intervention acceptability: At the 12-month follow-up, nominated supervisors in the intervention arm reported via CATI the acceptability of the Web-based menu planning program on a 5-point Likert scale (1=strongly agree; 5=strongly disagree), using items developed by the research team. The proportion reporting 2 or lower (agree or strongly agree) on each of these questions was calculated.

### Sample Size and Power Calculations

On the basis of pilot data (unpublished) with a standard deviation of 1.23, a sample of 27 services in the intervention and 27 services in the control would enable detection of a 0.96 (approximately 1) change in the mean number of food groups compliant between intervention and control groups at the 12-month follow-up (primary outcome) with 80% power and a 2-sided alpha of .05. From a population health perspective, increasing compliance with just 1 food group may contribute to important improvements in public health nutrition. For example, based on current data regarding food provision by childcare services in Australia [49], achieving compliance with guideline recommendations for vegetables would be equivalent to an increase of 60 grams (0.8 servings) per child, while compliance with discretionary foods would be equivalent to a decrease of 360 kilojoules (0.6 servings) per child [14]. Such improvements have been associated with important child health outcomes and reductions in disease risk [50,51].

### Statistical Analysis

All statistical analysis was undertaken using SAS 9.3 (SAS Institute Inc) [52] by a statistician blinded to group allocation. All statistical analyses were 2-tailed with an alpha value of .05. Service postcodes ranked in the top 50% of NSW according to the 2016 Socioeconomic Indices for Areas were classified as higher socioeconomic status [37]. Geographical characteristics of service locality were classified as either urban or rural according to the Australian Statistical Geography Standard [53]. Chi-square and *t* test analyses were used to compare characteristics of consenters and nonconsenters, and service and menu planner characteristics between intervention and control groups at baseline. The primary (mean number of food groups compliant with guidelines) and secondary menu outcomes (individual and all food group compliance with guidelines, and mean daily servings of individual food groups) were analyzed with generalized linear mixed models to account for repeated measures at the service level, as well as potential service level clustering effects for the mean daily servings of food groups analysis. All models included a random effect for service, as well as a group by time interaction to assess intervention effectiveness over the 3 time points (summarized as relative mean difference for the continuous measures and relative odds ratio [OR] for the categorical outcomes). All models assessed the relative difference in menu outcomes between the 2 groups from baseline to 3 months, as well as the relative difference from baseline to 12 months. For the primary and secondary outcomes, under an intention-to-treat framework, a complete case analysis was performed using all available data based on group allocation (without imputation), in addition to analysis using multiple imputation for missing data at follow-up undertaken using the MI procedure in SAS.

## Results

### Baseline Characteristics of Study Participants

**Figure 1:**
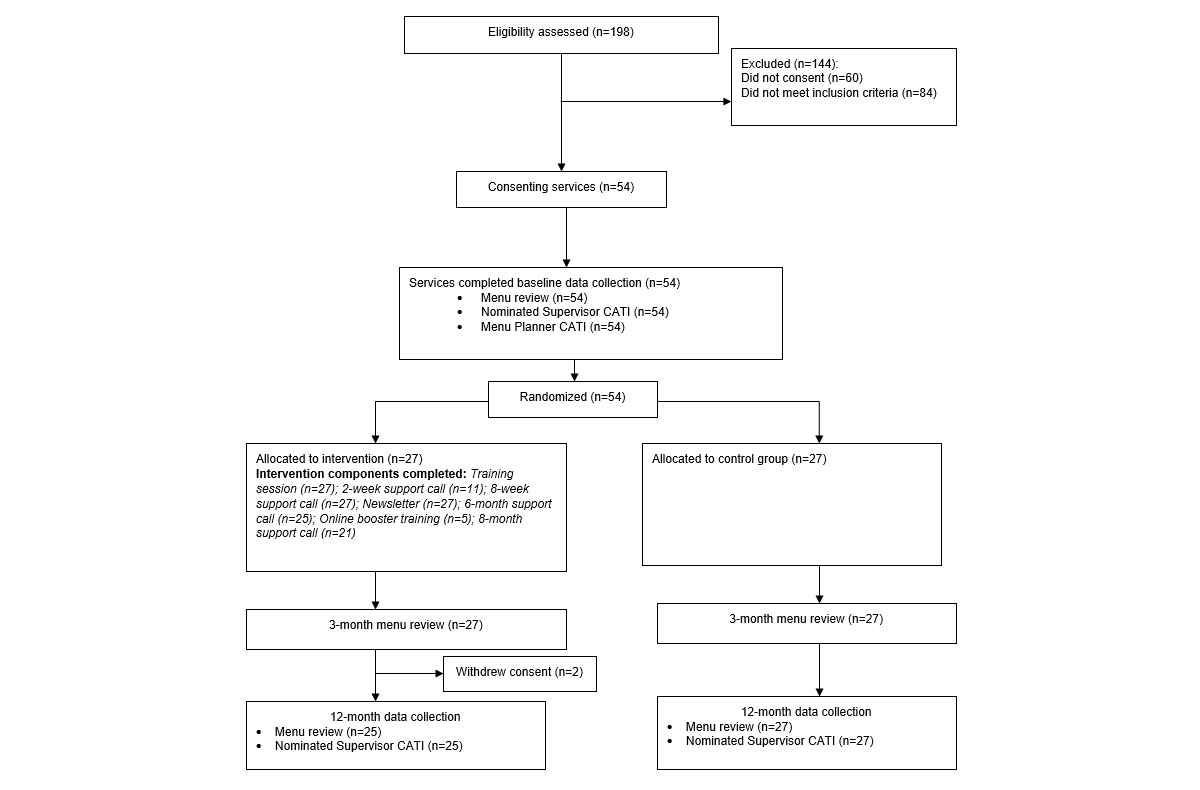
Consolidated Standards of Reporting Trials diagram. CATI: computer-assisted telephone interview.

Of the 252 long day care services, who were current clients of a single specific CCMS provider in the study region, 54 services declined to participate in the study before eligibility assessment. A total of 198 services were assessed for eligibility, with 42.4% (84/198) deemed ineligible, most commonly because of the inability of service staff to make menu planning decisions (28/84, 33%), and not providing meals and snacks to children (24/84, 29%); (Figure 1). Of the remaining 114 eligible services, 47.4% (54/114) provided consent to participate in the study. There were no significant differences in service area socioeconomic status or service geographic location between consenters and nonconsenters.

A total of 27 services were randomized to the intervention and 27 services to the control. Two intervention services withdrew from the study before the 12-month follow-up; 1 no longer prepared and provided meals and the other no longer wished to participate. Services in the control arm had a significantly higher proportion of menu planners with a university qualification (5/27, 19%) compared with services in the intervention (0/27, 0%; *P*=.02; Table 1).

**Table 1:** Baseline demographic characteristics of participating childcare service, menu planner and children.

Characteristics			Intervention (n=27)		Control (n=27)	
**Service**						
	**Area socioeconomic status (n=53), n (%)**					
		High socioeconomic status		17 (63)		15 (56)
		Low socioeconomic status		10 (37)		11 (41)
	**Geographic location (n=53), n (%)**					
		Urban (major cities)		24 (89)		19 (73)
		Rural (inner regional, outer regional, remote)		3 (11)		7 (27)
	Services with children of aboriginal background, n (%)			14 (52)		18 (67)
	Number of children attending each day, mean (SD)			49.8 (18.6)		45.0 (16.8)
	Hours open per day, mean (SD)			10.6 (0.5)		10.8 (0.7)
	Number of primary contact educators, mean (SD)			12.3 (9.8)		10.5 (4.5)
**Menu planner**						
	Age (years), mean (SD)			48.4 (10.4)		44.9 (10.5)
	**Qualifications, n (%)**					
		University qualification		0 (0)		5 (19)
		Technical and Further Education		8 (30)		14 (52)
		Registered training organizational course		12 (44)		7 (26)
		“On the job” training		7 (26)		8 (30)
		Commercial cooking qualification		7 (26)		6 (22)
	Years working as menu planner, mean (SD)			9.4 (8.6)		10.3 (8.9)

### Primary Outcome

#### Mean Number of Food Groups Compliant With Dietary Guidelines

Although an increase in the mean number of food groups compliant with dietary guidelines from baseline to follow-up was found for both intervention and control services, no significant differences between the groups were found at the 3-month follow-up (mean difference 0.52; 95% CI −0.35 to 1.39; *P*=.24; Table 2) or the 12-month follow-up (mean difference 0.26; 95% CI −0.61 to 1.14; *P*=.55; Table 3).

**Table 2:** Baseline and 3-month primary and secondary outcome menu compliance with dietary guidelines: Results for participating childcare services.

Measure			Intervention					Control					Complete case analysis^a^: Baseline versus 3 months				
			Baseline (n=27)		3 months (n=27)		Baseline (n=27)			3 months (n=27)		Relative effect size					
												Mean difference (95% CI)			Odds ratio (95% CI)		*P* value
Number of food groups compliant (n=6), mean (SD)			1.19 (1.33)		2.15 (1.90)		0.96 (1.13)			1.41 (1.15)		0.52 (−0.35 to 1.39)			—		.24
Compliance for all food groups (n=6), n (%)			0 (0)		1 (4)		0 (0)			0 (0)		—			—^b^		—
**Compliance with individual food groups, n (%)**																	
	Vegetables	1 (4)		6 (22)		1 (4)			4 (15)		—			1.65 (0.07 to 40.33)		.76	
	Fruit	7 (26)		11 (41)		8 (30)			5 (19)		—			4.33 (0.69 to 27.29)		.12	
	Cereals and breads	10 (37)		15 (56)		7 (26)			9 (33)		—			1.55 (0.29 to 8.42)		.61	
	Meat and alternatives	3 (11)		9 (33)		2 (7)			5 (19)		—			1.48 (0.14 to 15.42)		.74	
	Dairy and alternatives	8 (30)		9 (33)		7 (26)			11 (41)		—			0.59 (0.11 to 3.19)		.54	
	Discretionary	3 (11)		8 (30)		1 (4)			4 (15)		—			0.75 (0.05 to 12.21)		.84	

^a^Complete case analysis under an intention-to-treat framework—analysis using all available data for menu compliance for baseline and follow-ups in the group to which they were originally assigned.

^b^Statistical analysis could not be performed.

**Table 3:** Baseline and 12-month primary and secondary outcome menu compliance with dietary guidelines: Results for participating childcare services.

Measure			Intervention				Control				Complete case analysis^a^: Baseline vs 12 months						Overall *P* value
			Baseline (n=27)		12 months (n=25)		Baseline (n=27)		12 months (n=27)		Relative effect size						
											Mean difference (95% CI)		Odds ratio (95% CI)		*P* value		
Number of food groups compliant (n=6), mean (SD)			1.19 (1.33)		1.80 (1.55)		0.96 (1.13)		1.30 (1.10)		0.26 (−0.61 to 1.14)		—		.55		.5
Compliance for all food groups (n=6), n (%)			0 (0)		0 (0)		0 (0)		0 (0)		—		—^b^		—		—
**Compliance with individual food groups, n (%)**																	
	Vegetables	1 (4)		2 (8)		1 (4)		5 (19)		—		0.37 (0.01 to 10.82)		.56		.43	
	Fruit	7 (26)		11 (44)		8 (30)		8 (30)		—		2.46 (0.41 to 14.58)		.32		.28	
	Cereals and breads	10 (37)		8 (32)		7 (26)		5 (19)		—		1.21 (0.20 to 7.51)		.83		.87	
	Meat and alternatives	3 (11)		6 (24)		2 (7)		3 (11)		—		1.70 (0.14 to 20.56)		.68		.91	
	Dairy and alternatives	8 (30)		11 (44)		7 (26)		11 (41)		—		0.97 (0.18 to 5.18)		.97		.78	
	Discretionary	3 (11)		7 (28)		1 (4)		3 (11)		—		0.99 (0.06 to 17.29)		.99		.96	

^a^Complete case analysis under an intention-to-treat framework—analysis using all available data for menu compliance for baseline and follow-ups in the group to which they were originally assigned.

^b^Statistical analysis could not be performed.

### Secondary Outcomes

#### Compliance With Dietary Guidelines for All Food Groups

At 3 months, only 1 (1/27, 4%) service in the intervention arm was compliant with dietary guideline recommendations for all 6 food groups (Table 2). At the 12-month follow-up, no services in either group were compliant with dietary guidelines for all 6 food groups (Table 3). Statistical analysis could not be performed, given the inadequate values in all cells.

#### Individual Food Group Compliance With Dietary Guidelines

An increase in the proportion of services compliant with individual food groups from baseline to follow-up was found for both intervention and control services, for the majority of food groups (4 out of 6). However, no significant differences between groups were found at the 3-month (Table 2) or 12-month (Table 3) follow-up for any individual food group.

#### Mean Servings of Individual Food Groups

Exploratory analyses revealed that at the 3-month follow-up, menus from services in the intervention group provided significantly more mean daily servings of fruit, vegetables, dairy, and meat, and significantly reduced the number of times discretionary foods were provided compared with control (Table 4). At the 12-month follow-up, menus from intervention services provided significantly more mean daily servings of fruit and significantly less discretionary foods compared with control service menus (Table 5).

**Table 4:** Baseline and 3-month mean daily servings of individual food groups on the menu for participating childcare services.

Measure	Intervention^a^		Control^a^		Complete case analysis^b^: Baseline versus 3 months	
	Baseline (n=27), mean (SD)	3 months (n=27), mean (SD)	Baseline (n=27), mean (SD)	3 months (n=27), mean (SD)	Relative effect size	
					Mean difference (95% CI)	*P* value
Vegetables	1.72 (1.15)	2.23 (1.27)	1.96 (1.28)	2.05 (1.30)	0.41 (0.05 to 0.78)	.03
Fruit	1.09 (0.72)	1.28 (0.55)	1.30 (0.79)	1.02 (0.55)	0.47 (0.29 to 0.66)	<.001
Cereals and breads	2.75 (1.28)	3.00 (1.40)	2.75 (1.47)	2.70 (1.31)	0.30 (−0.10 to 0.71)	.14
Meat and alternatives	0.73 (0.46)	0.96 (0.55)	0.87 (0.58)	0.85 (0.50)	0.24 (0.09 to 0.40)	.003
Dairy and alternatives	1.17 (0.63)	1.26 (0.70)	1.31 (0.64)	1.18 (0.57)	0.21 (0.03 to 0.40)	.03
Discretionary (times)	0.62 (0.71)	0.33 (0.52)	0.70 (0.80)	0.64 (0.76)	−0.24 (−0.45 to −0.03)	.02

^a^Calculated from service mean daily servings data (5 days of data per service).

^b^Complete case analysis under an intention-to-treat framework—analysis using all available data for menu compliance for baseline and follow-up in the group to which they were originally assigned.

**Table 5:** Baseline and 12-month mean daily servings of individual food groups on the menu for participating childcare services.

Measure	Intervention^a^		Control^a^		Complete case analysis^b^: Baseline versus 12 months		Overall *P* value
	Baseline (n=27), mean (SD)	12 months (n=25), mean (SD)	Baseline (n=27), mean (SD)	12 months (n=27), mean (SD)	Relative effect size		
					Mean difference (95% CI)	*P* value	
Vegetables	1.72 (1.15)	2.04 (0.97)	1.96 (1.28)	2.12 (1.26)	0.14 (−0.23 to 0.51)	.45	.08
Fruit	1.09 (0.72)	1.30 (0.73)	1.30 (0.79)	1.27 (0.79)	0.21 (0.02 to 0.40)	.03	<.001
Cereals and breads	2.75 (1.28)	2.90 (1.42)	2.75 (1.47)	2.81 (1.59)	0.04 (−0.37 to 0.45)	.85	.28
Meat and alternatives	0.73 (0.46)	0.88 (0.39)	0.87 (0.58)	0.88 (0.63)	0.12 (−0.03 to 0.28)	.12	.01
Dairy and alternatives	1.17 (0.63)	1.21 (0.64)	1.31 (0.64)	1.24 (0.63)	0.10 (−0.09 to 0.29)	.29	.08
Discretionary (times)	0.62 (0.71)	0.23 (0.51)	0.70 (0.80)	0.63 (0.77)	−0.33 (−0.54 to −0.11)	.003	.008

^a^Calculated from service mean daily servings data (5 days of data per service).

^b^Complete case analysis under an intention-to-treat framework—analysis using all available data for menu compliance for baseline and follow-up in the group to which they were originally assigned.

No changes to the statistical significance of any outcomes were observed in the multiple imputation analyses, and as such these results are not reported.

#### Use of the Web-Based Menu Planning Program

At approximately 12-month follow-up, intervention services had logged into the Web-based menu planning program an average of 40.4 (SD 31.8) times, spending an average of 47.1 (SD 65.2) min in the program per login (Table 6).

**Table 6:** Use of the Web-based program among intervention services at the 12-month follow-up (N=25).

Measure	Mean (SD)	Median (IQR)
Number of times logged in	40.4 (31.8)	35.0 (16.0-52.0)
Number of times the menu was accessed	69.5 (54.7)	55.0 (31.0-107.0)
Number of times recipes were accessed	10.8 (11.3)	6.0 (4.0-13.0)
Number of recipes used	89.2 (119.2)	20.0 (1.0-140.0)
Number of times nutrition checklist was accessed	8.0 (14.2)	4.0 (2.0-6.0)
Number of times analytics was accessed	6.2 (6.1)	5.0 (2.0-6.0)
Time in program (hours)	38.8 (108.4)	13.3 (6.9-20.9)
Time per login (min)	47.1 (65.2)	34.9 (18.8-47.5)
Number of times helpdesk was contacted	0	—^a^

^a^Unable to be calculated.

#### Intervention Acceptability

Over 90% (23/25) of nominated supervisors reported the Web-based menu planning program to be useful with planning menus to meet dietary guidelines and 88% (22/25) would recommend the program to other childcare services (Table 7).

**Table 7:** Acceptability of the Web-based program reported by nominated supervisors in the intervention at the 12-month follow-up.

Measure (score ≤2 [agree or strongly agree])	Value, n (%)
The Web-based menu planning program was useful in my service to help staff with planning menus to meet the dietary guidelines.	23 (92)
Using the Web-based menu planning program improved my services performance in planning menus to meet the dietary guidelines.	22 (88)
Using the Web-based menu planning program is an acceptable method for assessing our services menu compliance with the dietary guidelines.	22 (88)
The children benefited from our service’s use of the Web-based menu planning program.	22 (88)
My service intends to continue to use the Web-based menu planning program to plan menus to meet the dietary guidelines.	21 (84)
I would recommend the Web-based menu planning program to other childcare services.	22 (88)

#### Delivery of Implementation Support

All 27 (27/27, 100%) intervention services were offered and completed a face-to-face training session in use of the Web-based menu planning program with a health promotion officer; 5 (5/27, 19%) services received a second training session because of technical issues (n=1); difficulties using the program (n=3), and staff returning from leave (n=1); 11 (11/27, 41%) menu planners received a brief support call 2 weeks following their training session (based on service needs) and 27 (27/27, 100%) received a support phone call at 8 weeks. All 27 services (27/27, 100%) were sent a study newsletter. A total of 25 (25/27, 93%) nominated supervisors received a support phone call at 6 months and 9 (9/27, 33%) menu planners received an online booster training session at 6 months (offer of training based on service needs). Finally, 21 (21/27, 78%) menu planners received a final support call at 8 months.

## Discussion

### Principal Findings

This study is the first RCT measuring the effectiveness of a Web-based menu planning program, linked to a CCMS system, in improving childcare service compliance with dietary guidelines. The study found that, despite being considered acceptable by childcare service staff, the intervention did not significantly improve childcare service menu or food group compliance with dietary guidelines compared with the control. However, significant increases in the servings of fruit, vegetables, dairy, and meat on the menu, and a significant reduction in the number of times discretionary foods were provided were observed at 3 months. At 12 months, a significant increase in servings of fruit and a significant reduction in the provision of discretionary foods was found. Such findings suggest that despite increases in the quantity of some healthy foods and a decrease in unhealthy (discretionary) foods provided on the menu, the Web-based intervention was not sufficiently effective to ensure children are provided with servings of food groups consistent with dietary guidelines for the setting. As foods provided in the home and other settings often fail to align with dietary guidelines [54], such findings are of concern.

The lack of a significant effect of the intervention on menu compliance with all food groups is similar to previous Australian studies in the childcare setting [17,19]. This suggests the achievement of a fully compliant menu in accordance with the current dietary guidelines for the setting is a sizeable challenge [55], and perhaps an unachievable goal for many childcare services at present. To be fully compliant with guidelines, services are required to provide adequate servings of each of the AGHE foods groups, and no discretionary foods, for every child in attendance on every single day. Reviews of public health program implementation more broadly suggest that implementation of more than 80% of recommended program elements is rarely achieved across a range of settings [56]. As such, continuous, incremental changes to practice may be more manageable, and over time may result in greater improvements in the provision of healthy food in childcare.

On measures of individual food group compliance, the ORs reported in this study at any time point (0.37-4.33) were generally smaller than those found in a previous randomized trial (1.19-17.83) which, using the same measure, found statistically significant improvements in compliance for fruit, meat, dairy, and discretionary foods [19]. In that 6-month face-to-face intervention, support for childcare service staff included securing executive support, 2 rounds of staff training and ongoing telephone support from an implementation support officer, provision of resources, and 2 rounds of audit and feedback from a dietitian. The findings may reflect a greater capacity of the more intensive face-to-face implementation support offered in the trial by Seward and colleagues to address a broader range of barriers to implementation (eg, environmental context). Such findings suggest the inclusion of additional implementation support strategies as an adjunct to the Web-based program, may be required in order for larger improvements in guideline implementation to be achieved. Future research testing this hypothesis is warranted.

Notwithstanding the lack of statistical significance between group effects on these measures, increases in compliance for all food groups and individual food groups for the control group were observed and were similar to those found in the intervention group. A possible explanation for this could be an increased awareness of the importance of healthy food provision in childcare in the external environment, other secular trends, or changes to childcare service accreditation requirements during the study period [57]. Alternatively, this may be the evidence of measurement reactivity or Hawthorne effect [58], in that the act of evaluating childcare service menus by external dietitians on multiple occasions within a 12-month period may lead to an increase in menu compliance with guidelines. To reduce the impact of any research reactivity effects, future studies should investigate alternate methods of measuring guideline implementation.

The exploratory analysis identified a statistically significant increase in the mean daily servings of food groups, in particular fruit, and a reduction in discretionary foods at both 3 months and 12 months among the intervention group, compared with the control. As the program focused on supporting services to make incremental changes to the quantities of healthy food groups provided on the menu via recipe substitution and modification, such improvements to servings are not surprising. In addition, the mean number of daily servings for some food groups (eg, fruit, breads and cereals, and dairy) was higher than the required minimum servings to be considered compliant with the guidelines, suggesting it is likely that services were compliant on some, but not all days of the week (as required for menu compliance). Assessments of any adverse impacts of the provision of foods above the recommended minimum on child-level outcomes or service outcomes (eg, increased waste) warrants investigation.

Among intervention services, there were high levels of acceptability and variable levels of use of the Web-based program (as evidenced by the large SDs and IQRs in program use data). Previous research has identified engagement with Web-based interventions to be associated with a range of health behaviors [59,60]. As such, research exploring perceived barriers and enablers to use of the program and identification of strategies to best support end-user engagement with the Web-based program is warranted.

### Limitations

The study had notable strengths including the design (RCT), rigorous evaluation approaches, and inclusion of theory-driven and evidence-based intervention and implementation support strategies. Limitations, however, were also present. Similar to previous trials within childcare services [61], the study yielded a moderate consent rate (47.4%). Although there were no significant differences in service area socioeconomic status or geographic location for consenters and nonconsenters, given the study was conducted within 1 state in Australia (NSW) with few Indigenous services, it is unclear whether these findings are generalizable nationally or internationally. Furthermore, despite randomization, services in the control arm had a significantly higher proportion of menu planners with a university qualification compared with the intervention services. It is possible that this may account for the improvement in menu compliance observed in the control arm. The findings report the overall effects of the intervention, which may mask differences in outcomes at the subgroup level. Future exploratory studies reporting findings from the trial will describe any differential effects by subgroups based on service locality (eg, service area socioeconomic status and geographic location), service characteristics (eg, size), or other contextual factors. Although the menu planning program was linked to a CCMS platform to increase uptake and integration into daily routines, the program was not viewable on the main child enrollments page that is accessed on a daily basis by childcare service staff. Integrating the Web-based menu planning program into the main CCMS platform of the software may reduce variability in service use of the program. Finally, the outcome relating to servings of individual food groups provided on the menu was not prospectively registered and should be interpreted with caution.

### Conclusions

The study is the first RCT measuring the effectiveness of a Web-based menu planning program to improve childcare service compliance with dietary guidelines in NSW, Australia. Findings indicate that the Web-based program was not effective in increasing the mean number of food groups compliant with dietary guidelines, nor the proportion of service menus compliant with dietary guidelines for all food groups and individual food groups. Despite this, significant improvements in the mean number of servings of healthy food groups and a reduction in the provision of discretionary foods provided on the menu were found. Future research should aim to reduce potential measurement reactivity or Hawthorne effects. Exploration of differing strategies in supporting uniform use of the Web-based program, and the implementation of dietary guidelines, among childcare services is warranted to ensure potential public health benefits are achieved.

## Appendix

Multimedia Appendix 1 Summary of the 12-month intervention components.

Multimedia Appendix 2 CONSORT-EHEALTH checklist (V 1.6.1).

## Abbreviations

AGHE: Australian Guide to Healthy Eating
CATI: computer-assisted telephone interview
CCMS: Childcare Management Software
CCNSW: Cancer Council NSW
NHMRC: National Health and Medical Research Council
NSW: New South Wales
OR: odds ratio
RCT: randomized controlled trial
