# Supplementary material for: Effectiveness of a Web-Based Menu-Planning Intervention to Improve Childcare Service Compliance With Dietary Guidelines: Randomized Controlled Trial
Source: J Med Internet Res. 2020 Feb 4;22(2):e13401. doi: 10.2196/13401 (PMC7055768; doi:10.2196/13401)
Supplement: Multimedia Appendix 1 [file jmir_v22i2e13401_app1.docx]

Multimedia Appendix 1. Summary of the 12-month intervention components.

| Intervention component according to protocol | Intervention strategy according to ERIC^a^ | Details |
| --- | --- | --- |
| Web-based menu planning program with decision support [28] | *Audit and feedback:* Collect and summarize performance data over a specified time period and give it to childcare providers to monitor, evaluate, and modify behavior. | - Actor: Web-based program - Action: Undertake menu planning by adding recipes, meals, snacks, and beverages into the Web-based program. The program calculates the total servings within each food group for each meal per child and per day. Nutritional analysis within the program is underpinned by a nutrition database adapted from a publicly accessible Australian food database [38]. Development of the nutritional database has been described elsewhere [34]. - An automated audit and feedback cycle was a core feature of the program [39]. Once a menu was entered, the program alerted menu planners and supervisors in real time where the menu was not compliant with the Caring for Children Guidelines (best practice dietary guidelines for the sector in NSW) [15] and included supporting Web-based resources to assist the user to adapt menus accordingly. Any modifications made to the menu triggered a real-time update of the menu compliance. Automated feedback reports of compliance were also generated, providing specific suggestions on how to ensure compliance for each food group (nutrition checklist). Information on child allergies or dietary requirements appeared as notifications to assist menu planners in considering individual child dietary needs. - Menu compliance over time was also provided in bar chart and line graph form within the program, according to overall menu, and by individual food groups to allow service tracking of improvements in menu compliance over time (analytics). - Targets: Menu planners - Temporality: Immediate commencement within intervention period - Dose: Ongoing over the intervention period |
| Web-based resource: | *Distribute educational materials:* Distribute educational materials (including guidelines, manuals, and toolkits) in person, by mail, and/or electronically. | - Actor: Web-based program - Action: A searchable recipe database, including information on how foods and recipes contribute to food group recommendations was provided within the program. Recipes were taken from the dietary guidelines for the sector [15], as well as additional recipes developed by the research team, and had the ability to be imported into service’s current menus. The recipe database had the ability to filter by meal time and food group servings required to enable timely and accurate modification of the menu. - Additional resources tailored to target known barriers to menu planning were included within the program (eg, useful tips about menu planning and serving sizes, sample menus, and assistance with using the Web-based program). Manuals and video resources outlining key features of the program were also accessible with the program. - Targets: Menu planners - Temporality: Immediate commencement within intervention period - Dose: Ongoing over the intervention period |
| Web-based reminders [40] | *Remind providers:* Develop reminder systems designed to help childcare providers to recall information and/or prompt them to implement the guidelines. | - Actor: Web-based program - Action: Fortnightly prompts to access the Web-based program were displayed to services on their CCMS^b^ dashboard when their current menu was not compliant with guidelines, insufficient information was entered into the program to calculate compliance, or their new menu cycle was due to be entered. - Targets: Menu planners, supervisors - Temporality: Immediate commencement within intervention period - Dose: Fortnightly over the intervention period |
| Communication strategies and managerial support [39,41] | *Involve executive boards:* Involve existing governing structures (eg, boards of directors and medical staff boards of governance) in the implementation effort, including the review of data on implementation processes.  *Involve patients/consumers and family members:* Engage or include patients/consumers and families in the implementation effort. | - Actor: Web-based program - Action: Via a Web-based parent portal, weekly menus and reports describing service compliance with dietary guidelines were displayed to parents of children attending care, a requirement of accreditation standards. Automated daily and weekly reports of compliance with dietary guidelines were also printable for display in the service. These reports also featured tips to help improve compliance for specific food groups. Positive messages viewable to all staff accessing the CCMS dashboard were displayed when improvements to the menu had been made. - To facilitate support for healthy meal provision, supervisors were encouraged to provide allocated time for menu planners to use the Web-based system and discuss improvements to the menu. - Targets: Menu planners, supervisors - Temporality: Immediate commencement within intervention period - Dose: Daily over the intervention period |
| Training and support to use the program [45] | *Conduct educational outreach visits:* A trained person (health promotion officer with nutrition and dietetic qualifications) meets with childcare providers in their practice settings with the intent of changing their behavior to implement the guideline.  *Provide ongoing consultation:* Provide ongoing consultation with one or more experts in the strategies used to support implementing the guidelines.  *Centralize technical assistance:* Develop and use a centralized system to deliver technical assistance focused on implementation issues. | - Actor: Health promotion officer with nutrition and dietetic qualifications - Action: A face-to-face training session with a health promotion officer, with nutrition and dietetic qualifications, and with extensive experience using the program, was provided to supervisors and menu planners. Training included information regarding the dietary guidelines for the sector, a demonstration of the Web-based program and supporting resources, and an opportunity to answer any queries. Action planning and goal setting with staff was undertaken with the goal of facilitating integration of the program into existing roles and service procedures [42,43]. - Services received telephone support calls by a health promotion officer, tailored to their engagement with the program and menu compliance with guidelines. - Services were sent 1 newsletter via email and within the CCMS program, containing ideas to increase menu compliance and tips for using the program. - Services without a current menu entered in the program and those who had not improved in menu compliance were offered an online booster training session [44]. - Online support was also available from the technical and nutritional support teams of the CCMS provider via an Web-based portal (help desk) already used by the services for all other IT queries. - Targets: Menu planners, supervisors - Temporality: 3-hour face-to-face training session within 4 weeks of intervention commencement; 5- to 30-min telephone support calls provided at 2 weeks, 8 weeks, 6 months, and 8 months; newsletter provided at 4 months; 15- to 60-min online booster training sessions provided at 6 months - Dose: Tailored to service needs—one-off face-to-face training session; up to 4 telephone support calls over 8 months; one-off newsletter; one-off online booster training session |
| Additional resources (portable computer tablet) | *Change equipment:* Evaluate current configurations and adapt, as needed, the equipment (eg, adding equipment) to best accommodate the targeted innovation | - Actor: Health promotion officer with nutrition and dietetic qualifications - Action: Services were provided with a Wi-Fi-enabled computer tablet (Samsung Galaxy) to maximize integration of the Web-based program into usual practice and provide the menu planner with portable access. - Targets: Menu planners, supervisors - Temporality: Computer tablet provided at initial training session - Dose: One-off computer tablet |

^a^Definitions derived from the Expert Recommendations for Implementing Change (ERIC) project.

^b^CCMS: Childcare Management Software.
